# Left ventricular dyssynchrony measured by cardiovascular magnetic resonance-feature tracking in anterior ST-elevation myocardial infarction: relationship with microvascular occlusion myocardial damage

**DOI:** 10.3389/fcvm.2023.1255063

**Published:** 2023-10-10

**Authors:** Zheng Sun, Yu Wang, Yingying Hu, Fang Wu, Nan Zhang, Zhi Liu, Jie Lu, Kuncheng Li

**Affiliations:** ^1^ Department of Radiology and Nuclear Medicine, Xuanwu Hospital, Capital Medical University, Beijing, China; ^2^ Beijing Key Laboratory of Magnetic Resonance Imaging and Brain Informatics, Beijing, China; ^3^ School of Biomedical Engineering, Capital Medical University, Beijing, China; ^4^ Department of Radiology, The Peking University International Hospital, Beijing, China; ^5^ Department of Emergency, Xuanwu Hospital, Capital Medical University, Beijing, China

**Keywords:** cardiovascular magnetic resonance-feature tracking, ST-elevation myocardial infarction, microvascular occlusion, myocardial dyssynchrony, strain

## Abstract

**Objectives:**

Cardiovascular magnetic resonance-feature tracking (CMR-FT) enables quantification of myocardial deformation and may be used as an objective measure of myocardial involvement in ST-elevation myocardial infarction (STEMI). We sought to investigate the associations between myocardial dyssynchrony parameters and myocardium damage for STEMI.

**Methods:**

We analyzed 65 patients (45–80 years old) with anterior STEMI after primary percutaneous coronary intervention during 3–7 days [observational (STEMI) group] and 60 healthy volunteers [normal control (NC) group]. Myocardial dyssynchrony parameters were derived, including global and regional strain, radial rebound stretch and displacement, systolic septal time delay, and circumferential stretch.

**Results:**

CMR characteristics, including morphologic parameters such as left ventricular ejection fraction (LVEF) (45.3% ± 8.2%) and myocardium damage in late gadolinium enhancement (LGE) (19.4% ± 4.7% LV), were assessed in the observation group. The global radial strain (GRS) and global longitudinal strain (GLS) substantially decreased in anterior STEMI compared with the NC group (GRS: 19.4% ± 5.1% vs. 24.8% ± 4.0%, *P* < 0.05; GLS: −10.1% ± 1.7% vs. −13.7% ± 1.0%, *P* < 0.05). Among 362 infarcted segments, radial and circumferential peak strains of the infarcted zone were the lowest (14.4% ± 3.2% and −10.7% ± 1.6%, respectively). The radial peak displacement of the infarct zone significantly decreased (2.6 ± 0.4 mm) (*P* < 0.001) and manifested in the circumferential displacement (3.5° ± 0.7°) in the STEMI group (*P* < 0.01). As microvascular occlusion (MVO) was additionally present, some strain parameters were significantly impaired in LGE^+^/MVO^+^ segments (radial strain [RS]: 12.2% ± 2.1%, circumferential strain [CS]: −9.6% ± 0.7%, longitudinal strain [LS]: −6.8% ± 1.0%) compared to LGE^+^/MVO^−^ (RS: 14.6% ± 3.2%, CS: −10.8% ± 1.8%, LS: −9.2% ± 1.3%) (*P* < 0.05). When the extent of transmural myocardial infarction is greater than 75%, the parameter of the systolic septal delay (mean, 148 ms) was significantly reduced compared to fewer degrees of infarction (*P* < 0.01).

**Conclusion:**

In anterior STEMI, the infarcted septum swings in a bimodal mode, and myocardial injury reduces the radial strain contractility. A more than 75% transmural degree was the septal strain-contraction reserve cut-off point.

## 1. Introduction

Acute anterior myocardial infarction can cause myocardial stunning or motion abnormalities, resulting in decreased systolic function and negatively affecting global left ventricular (LV) function (1–3). Prinzen's study has first shown the relation between the electrical and mechanical activation sequence at the LV anterior wall infarction. The compensatory overactivity of the left ventricle's lateral wall worsens the poor prognosis caused by the lack of synchrony between the septal and remote lateral walls (4). Research has indicated that long-term abnormal interventricular septal motion can cause left bundle branch block (LBBB) (5–7). This occurs as a result of unusual activity in the septum, which is identified by significant rapid pre-ejection contractions, the movement of the septum towards the left, immediately followed by lengthening again (rebound stretch), and a contradictory motion of the septum swinging towards the right (8). However, the rebound extension occurs due to the delayed triggered compensatory contraction of the remote myocardium (9, 10). Studies using model simulations have shown that decreased contractility of the lateral wall of the left ventricle results in a reduction in the rebound stretch of the interventricular septum, which in turn leads to myocardial remodeling with positive effects (11). The extent of anterior infarction and its impact on the motion of peri-infarcted and remote myocardium remains uncertain. Since the septal function is critical to the global LV function and prognosis, we suggest further refining global and segmental septal viability analysis.

Anterior myocardial infarction assessment based on cardiovascular magnetic resonance imaging (CMR) has significant predictive value (12). Furthermore, recent research has concentrated on anomalies in deformation and motion, in addition to the size of myocardial infarction. Thanks to the advancement in CMR-feature tracking (CMR-FT) technology, strain can enhance the conventional cardiac function measure known as ejection fraction (EF), as it has certain drawbacks associated with its volumetric characteristics, limited reproducibility, and inability to indicate regional LV function (13, 14). Strain can provide different spatial components of contractile function in radial strain (RS), circumferential strain (CS), and longitudinal strain (LS) directions, both globally and regionally (15). Moreover, when ST-elevation myocardial infarction (STEMI) patients are accompanied by microvascular occlusion (MVO), their prognosis worsens, and the likelihood of reinfarction increases significantly. Hence, it is advisable to employ efficient detection of MVO and conduct an early evaluation of risks to minimize complications post-myocardial infarction. Strain in the context of acute STEMI can offer a non-invasive approach to assess myocardial tissue characteristics by combining morphological and functional imaging, serving as a compelling substitute evaluation marker.

Studies conducted over a long-term follow-up have revealed that approximately 33% of patients with LBBB do not encounter septal swing, indicating that the motion of the interventricular septum is influenced not only by the cardiac conduction factor but also by stronger myocardial self-compensation (6). Considering that the pre-ejection reduction and rebound stretch of the septum rely on the active contraction of the septum and remote lateral walls, we propose that the balance of strain between these two regions of the myocardium plays a vital role in influencing the movement of the septum. To examine this hypothesis, we assessed the infarcted and peri-infarcted myocardium, as well as the size of the infarction and the extent of injury, to investigate if any changes in septal function would affect the balance of strain.

This study aimed to characterize myocardial strain alterations by CMR-FT for patients with anterior STEMI. Simultaneously, our intention was to investigate the connections between infarcted, peri-infarcted, and non-infarcted myocardium in aspect of segmental mechanical dyssynchrony, systolic delay in the septum, and the stretching of radial and circumferential rebound strain.

## 2. Materials and methods

### 2.1. Study population

From January 2019 to May 2022, a total of 65 individuals diagnosed with anterior ST-elevation myocardial infarction (STEMI) were enrolled from the cardiology department at Xuanwu Hospital of Capital Medical University. There were 60 healthy volunteers in the normal control (NC) group. The normal control participants were enrolled with a negative cardiac history, without known cardiovascular risk factors, not on any medications, with a normal electrocardiogram and echocardiography, and normal cardiac MRI (16). As per the guidelines of the European Society of Cardiology (ESC) (17), patients aged between 45 and 80 years were included if they had symptoms lasting less than 12 h, ST-segment elevation of at least 0.1 mV in 2 consecutive leads on an electrocardiogram (ECG), or new-onset LBBB. Exclusion criteria included individuals who had previously experienced a heart attack or undergone coronary artery bypass grafting surgery, those who had suffered a cardiac arrest outside of a hospital and required cardio pulmonary resuscitation (CPR), individuals who were in cardiogenic shock, those who had received fibrinolysis within 72 h of presentation, or those who had contraindications to CMR imaging.

The study was approved by the Ethics Committee of Xuanwu Hospital of Capital Medical University (D2014044). According to the Declaration of Helsinki, written informed consent was obtained from each subject before enrollment.

### 2.2. CMR acquisition

Clinical imaging systems (Magnetom Verio 3.0T, Siemens Healthcare, Erlangen, Germany) were utilized for CMR studies conducted within 3–7 days following the occurrence of myocardial infarction. After thorough examination and evaluation, a series of imaging sequences were performed to capture detailed images of the left ventricle. These included a T2-weighted short-tau inversion recovery (T2w-STIR) sequence, a cine sequence using contrast enhancement steady-state free precession (CE-SSFP), and a late gadolinium enhancement (LGE) sequence. All sequences were acquired in a breath-held manner and covered the entire left ventricle including the four-chamber, two-chamber, and all short-axis views, ensuring accurate and comprehensive results. CE-SSFP was acquired immediately after the onset of contrast media administration, and LGE imaging was acquired after a minimum of 10 min following the intravenous administration of 0.2 mmol/kg of a contrast agent containing gadolinium (Magnevist, Bayer Healthcare, USA). Supplementary Appendix Table S1 contains information regarding the imaging sequences.

### 2.3. CMR image analysis

**Figure 1:**
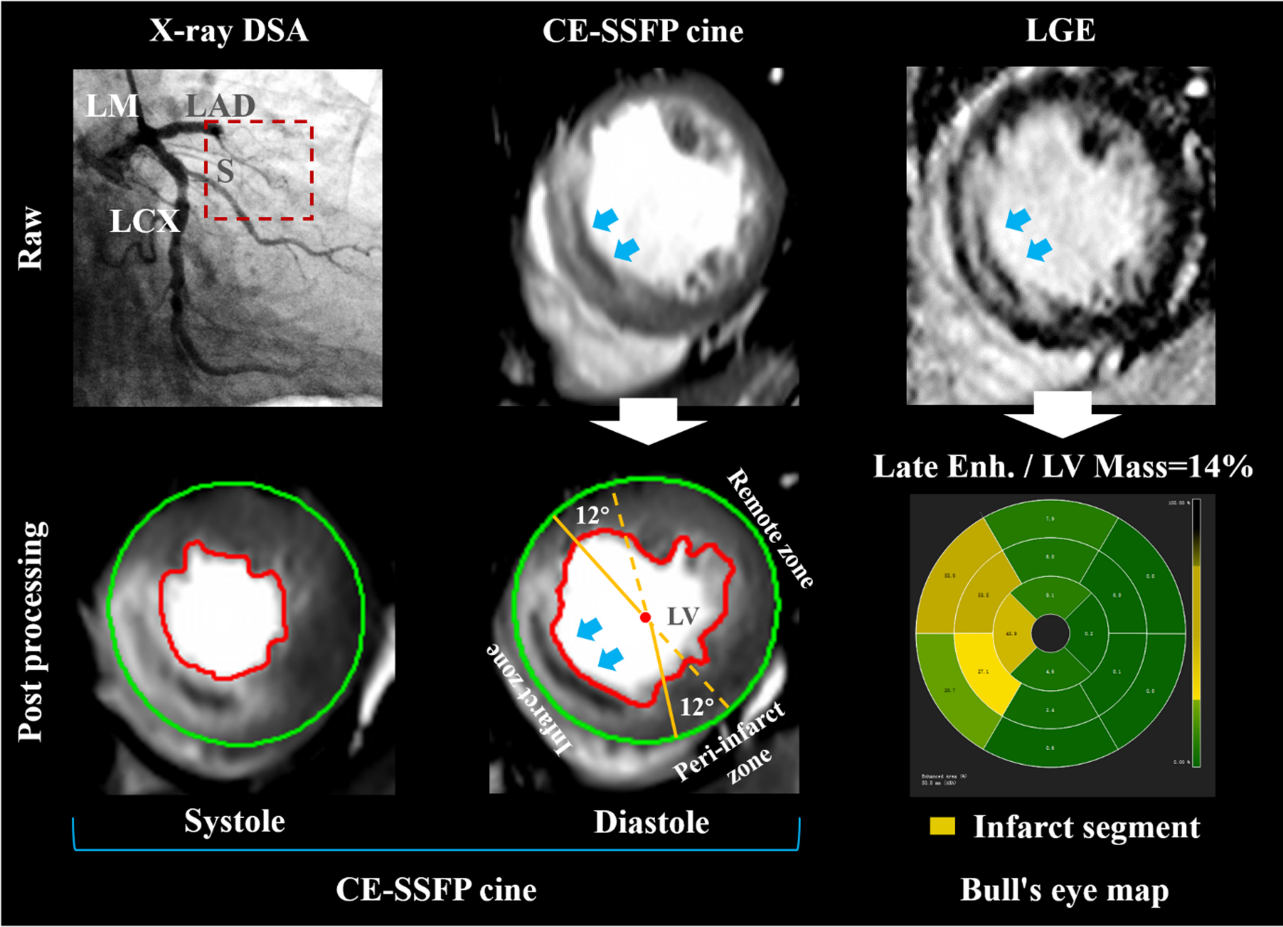
Delineation of MVO and peri-infarct zones. A 73-year-old patient with left anterior descending artery (LAD) occlusion was diagnosed by digital subtraction angiography (DSA) (upper row on the left). The red box with a dotted line refers to occlusive vessels. In CMR CE-SSFP cine images, epicardial and endocardial borders are delineated by green and red borders, respectively. The area of established microvascular occlusion (MVO) infarction (Blue arrows point to the dark area surrounded by the hyperintensity infarction zone and located at the same position within the cardiac wall on CE-SSFP cine image during the cardiac cycle.) was taken from late gadolinium enhancement (LGE) images. Patients without visible peri-infarct myocardium had strain measurements in the infarcted and remote zones and separately in a 12° sector circumferentially adjacent to either side of the infarct (centre of bottom row). The bull's eye map showed segmental analysis within the 16-segment model (bottom row on the right). DSA, digital subtraction angiography; LAD, left anterior descending artery; LGE, late gadolinium enhancement; LV, left ventricular; LCX, left circumflex artery; LM, left main; MVO, microvascular occlusion; S, branch of the interventricular septum.

YYH and FW, with 11 and 9 years of cardiovascular MR expertise respectively, analyzed the images offline using CVI^42^ [version 5.12.1 (1686), Circle Cardiovascular Imaging Inc., Calgary, Alberta, Canada]. In the post-processing of defining the left ventricular contour, manually comparing the CE-SSFP and LGE sequences can effectively eliminate the influence of strong endocardial signals caused by sluggish blood flow. CE-SSFP cine images were used to analyze LV volumes (in milliliters), EF (as a percentage), EDV (in milliliters), and ESV (in milliliters). T2w-STIR was employed to identify the myocardium affected by edema surrounding the infarct area in the early stages after STEMI. Additionally, CE-SSFP cine was utilized to ascertain the peri-infarct zone and the myocardium unaffected by the infarction, known as remote non-infarcted myocardium (RNM) (18). The RNM had a reference region of interest (ROI) established by applying the criterion of mean +2 standard deviation (SD) on CE-SSFP cine to define edema-based myocardium as a percentage of LV mass (19), and the criterion of mean +5 standard deviation (SD) on LGE to define an infarcted zone, which also included any hypodense core (20). Figure 1 depicted the peri-infarct zone as myocardium surrounding the infarcted myocardium, exhibiting hyperintensity on CE-SSFP cine images. Areas of interest were manually transferred between images by using software (CVI^42^), as described in previous studies (20). To prevent overestimation of the infarct size (21), the RNM was isolated from the peri-infarct area and remained unaffected. The size of myocardial infarction was regionally reported as a proportion of the entire tissue per wall and globally as a proportion of the entire tissue in the left ventricle (%LV mass). The segmental hyperintensity signal was categorized into four grades based on the extent of transmural injury: minimal or no (0%–25%), moderate (26%–50%), intense (51%–75%), and fully transmural (76%–100%). Every reader attributed it according to the 16-part pattern established by the American Heart Association. MVO was characterized by a black region surrounding LGE, located within the endocardium and indicating a no-reflow area. Moreover, the segments were visually categorized as segments exhibiting LGE and extra MVO (LGE^+^/MVO^+^), segments showing LGE but no MVO (LGE^+^/MVO^−^), or segments without any enhancement.

### 2.4. Strain analysis

The application of CMR-FT is possible on a standard CE-SSFP cine sequence without the need for a dedicated acquisition. The complex post-processing procedure involved the following steps: (i) tracing the endocardial and epicardial borders of the left ventricle in images taken from the four-chamber, two-chamber, and all short-axis views to segment the myocardium; (ii) identifying anatomical features along the myocardial boundaries in the cine image obtained from CE-SSFP and defining regions of interest (ROI) around these locations, which were then tracked throughout the cardiac cycle by searching for the most similar area in the subsequent image; (iii) determining the displacements of the myocardial tissues, including the borders, in each subsequent frame using a gradient-based optical flow method. To be more specific, the movement of myocardial segments can be assessed by creating small square windows around a characteristic on the initial image and then locating the greyscale pattern that closely resembles it on the subsequent image (22). Strain values from 2D strain analysis were collected for all studies, both global and regional, and exported for statistical analysis.

**Figure 2:**
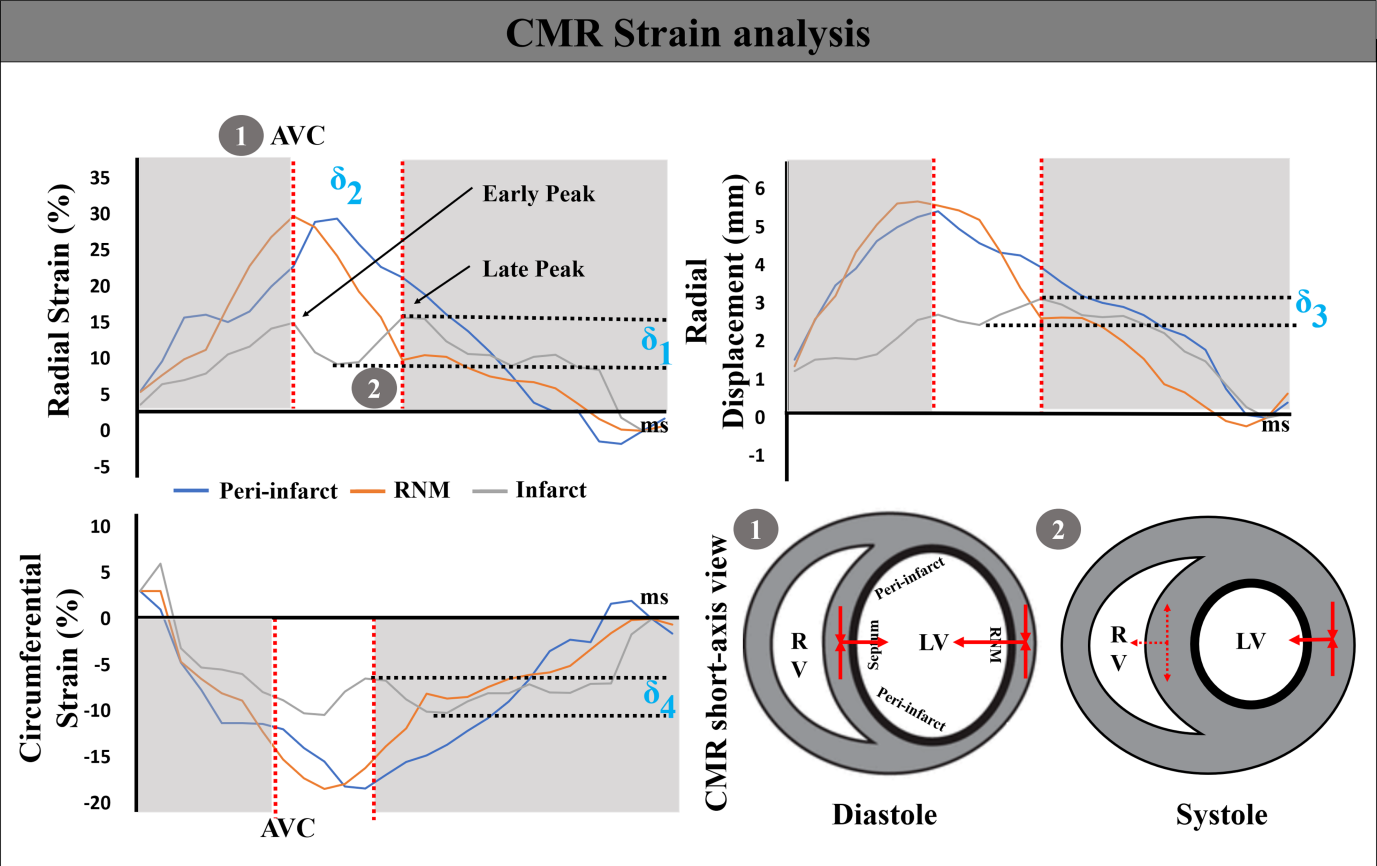
CMR strain analysis. The strain traces delineate the ventricular deformation. A double-peaked septal (gray), single-peaked remote non-infarcted myocardium (orange), and a pre-stretched lateral wall (blue) radial strain curve. δ1, δ2, δ3, and δ4 represent the degree of radial rebound of the septum, systolic septal delay, radial rebound displacement, and the circumferential strain stretch, respectively. Concomitant mechanical left ventricular (LV) events, including septal rebound stretch, are indicated with numbers. After the aortic valve closes, the interventricular septum is aligned with the direction of remote non-infarcted myocardial motion (number 1). However, as the left ventricle enters the systole, there is a reverse movement of the interventricular septum (number 2). AVC, aortic valve closure; RV, right ventricle.

Subsequently, five subsets of strain parameters were evaluated. First, global RS, CS, and LS analyses were conducted. Negative values were transformed into absolute numbers. Furthermore, the peak strain, time to peak, and peak displacement were performed by the utilized as segment levels in the infarcted zone, peri-infarct zone, and RNM. Next, the highest remote myocardial strain was established based on the timing of aortic valve closure (AVC), and then the peak values in the surrounding and damaged tissue were monitored. The radial rebound stretch (RRS) of the infarcted area was calculated based on the estimation shown in Figure 2 (9):

(1) $$\delta \_1=\mathrm{RR}S_{\mathrm{infarcted}\_\mathrm{late}\_\mathrm{peak}}-\mathrm{RR}S_{\mathrm{infarcted}\_\mathrm{valley}}\;(\text{\%})$$

Additionally, dyssynchrony was assessed by calculating the time interval between an initial peak and a subsequent peak in the infarcted septal segment (rebound of radial contraction in the septum):

(2) $$\delta \_2=\mathrm{Tim}e_{\mathrm{infarcted}\_\mathrm{early}\_\mathrm{peak}}-\mathrm{Tim}e_{\mathrm{infarcted}\_\mathrm{late}\_\mathrm{peak}}\;(\mathrm{ms})$$

The measurement of the systolic rebound stretch of the septum determined the lack of coordination between the septal radial rebound displacement (RRD) and CS stretch difference. They were estimated as *δ*3 and *δ*4:

(3) $$\delta \_3=\;\mathrm{RR}D_{\mathrm{infarcted}\_\mathrm{late}\_\mathrm{peak}}-\mathrm{RR}D_{\mathrm{infarcted}\_\mathrm{valley}}\;(\mathrm{mm}\;\mathrm{or}{\;}^{\circ })$$

(4) $$\delta \_4\;=\;-\;|CS_{\mathrm{infarcted}\_\mathrm{valley}}-CS_{\mathrm{infarcted}\_\mathrm{late}\_\mathrm{peak}}|\;\;(\text{\%})$$

### 2.5. Statistical analysis

Mean ± SD or median (IQR) were used to express continuous variables and their distribution normality was assessed using the Shapiro–Wilk test. The means and medians of two variables were compared using the appropriate methods, including the Student's *t*-test and Mann–Whitney *U* test. To compare multiple groups, we conducted an analysis of variance and Kruskal–Wallis *H* test. A two-tailed *P*-value < 0.05 was chosen as the threshold for statistical significance. The reproducibility of strain analysis was assessed using the Intraclass Correlation Coefficient (ICC). The ICC of 0.75 or greater was considered excellent; 0.75–0.40 as moderate; and less than 0.40 as poor. Standard statistical software SPSS (version 19.0, Chicago, IL, USA) was used for all analyses.

## 3. Results

### 3.1. Study population

The study protocol was completed by 65 patients and 60 volunteers. The average age of the patients was 64.7 ± 6.9 years, with 56 (86%) being male. No significant disparities in heart rate and systolic blood pressure were observed between the STEMI cohort and the NC group (*P* > 0.05). The CMR results showed that the patients' end-diastolic volume (EDV) and left ventricular ejection fraction (LVEF) were notably decreased compared to the NC group (70.1 ± 8.8 ml vs. 78.3 ± 12.6 ml; 45.3% ± 8.2% vs. 67.1% ± 7.8%, *P* < 0.05). The ESV of the patients rose (49.4 ± 7.7 ml compared to 33.8 ± 12.2 ml, with a significance level of *P* < 0.05). Cardiac magnetic resonance imaging with late gadolinium enhancement (CMR-LGE) was conducted on every patient diagnosed with STEMI, revealing that the average size of the infarct accounted for approximately 19.4% ± 4.7% LV mass. At the individual level, all patients exhibited LGE, and an additional presence of MVO was observed in 33 (51%) patients. Table 1 displays the general baseline characteristics.

**Table 1:** Demographic and clinical characteristics of the study population.

Variable	STEMI (*N* = 65)		NC group (*N* = 60)	*P*-value
	MVO^+^ (*N* = 33)	MVO^−^ (*N* = 32)		
Clinical characteristics				
Age (years)	65.4 ± 6.8	62.1 ± 7.2	64.1 ± 8.2	0.57
Male *n* (%)	29 (88)	27 (84)	39 (65)	**<0**.**05**
BMI (kg/m^2^)	29.1 ± 5.4	26.5 ± 2.1	27.5 ± 3.7	0.86
Heart rate (min^−1^)	66 ± 5	68 ± 7	66 ± 8	0.55
Systolic BP (mm Hg)	128 ± 10	133 ± 8	132 ± 10	0.65
Diastolic BP (mm Hg)	62 ± 10	67 ± 6	81 ± 11	**<0**.**05**
Diabetes *n* (%)	21 (64)	13 (41)	11 (18)	**<0**.**05**
Hypertension *n* (%)	13 (39)	17 (53)	21 (35)	**<0**.**05**
Smoke *n* (%)	27 (82)	26 (81)	49 (82)	0.44
Culprit artery				
LAD *n* (%)	31 (94)	28 (88)	–	–
LCX *n* (%)	0	0	–	–
RCA *n* (%)	2 (6)	4 (12)	–	–
CMR characteristics				
Day of baseline CMR (day)	6 ± 1	6 ± 1	–	–
Door to balloon time (h)	44 ± 7	49 ± 8	–	**0**.**65**
End-diastolic volume (ml)	70.5 ± 8.7	69.8 ± 9.1	78.3 ± 12.6	**<0**.**05**
End-systolic volume (ml)	53.4 ± 6.6	46.2 ± 8.0	33.8 ± 12.2	**<0**.**05**
LVEF (%)	41.2 ± 9.2	46.3 ± 5.4	67.1 ± 7.8	**<0**.**05**
Infarct size (%LV mass)	21.3 ± 3.2	16.4 ± 3.7	–	–
Dimensions end-diastole (mm)				
Septum	15.5 ± 0.9	14.9 ± 1.2	10.9 ± 1.1	**<0**.**05**
Remote	12.1 ± 1.1	12.5 ± 0.9	10.4 ± 0.8	0.78

Values are presented as mean ± SD, *n* (%). The *P-*values in bold are statistically significant. BMI, body mass index; BP, blood pressure. MVO^+^, MVO-positive; MVO^−^, MVO-negative.

### 3.2. Assessment of segmental strain parameters

According to strain analysis, the STEMI group showed a significant decrease in global radial strain (GRS) and global longitudinal strain (GLS) compared to the NC group. The GRS was 19.4% ± 5.1% in the STEMI group, while it was 24.8% ± 4.0% in the NC group (*P* < 0.05). Similarly, the GLS was −10.1% ± 1.7% in the STEMI group, whereas it was −13.7% ± 1.0% in the NC group (*P* < 0.05). Simultaneously, there was an increase in the global circumferential strain in the STEMI group, although the variation was not statistically significant (−17.6% ± 1.8% vs. −18.7% ± 2.2%, *P* > 0.05).

Sequentially, a total of 1,040 segments were studied in the STEMI group, while the NC group had 960 segments examined on a segmental basis. There was a total of 362 segments in the infarct zone, accounting for 35%. The peri-infarct zone displayed 390 segments, representing 38% of the total. Additionally, the RNM displayed 288 segments, making up 28%. Table 2 demonstrates the varying degrees of alteration in segmental strain values observed in the infarcted zone, peri-infarct zone, and RNM. Among the various wall ranges evaluated, the infarcted zone exhibited the least radial strain peak (14.4% ± 3.2%), with approximately half of the radial strain peak observed in the peri-infarct zone (26.9% ± 4.8%) or the non-infarcted myocardium located remotely (28.7% ± 3.9%) (*P* < 0.01). In the STEMI group, the radial strain of the RNM exceeded that of the anterior segments (25.1% ± 4.1%) and the RNM (24.8% ± 3.0%) in the NC group, with a significance level of *P* < 0.01. In the STEMI group, the infarcted zone had the lowest circumferential peak strain (−10.7% ± 1.6%), but there was no difference in the peri-infarct and RNM compared to the NC group (*P* > 0.01). Furthermore, the longitudinal peak strain of the infarct region (−8.9% ± 1.2%), peri-infarct region (−9.6% ± 1.4%), and RNM far from the infarcted area (−11.4% ± 1.1%) in the STEMI group exhibited a decrease compared to the corresponding segments in the NC group (*P* < 0.01).

**Table 2:** Segmental strain parameters analysis.

Variable	STEMI			NC		*P*-value
	Infarcted zone (*N* = 362)	Peri-infarct zone (*N* = 390)	RNM (*N* = 288)	Anterior (*N* = 480)	RNM (*N* = 480)	
Peak strain (%)						
Radial	14.4 ± 3.2	26.9 ± 4.8*	28.7 ± 3.9*	25.1 ± 4.1*^,^^¶^	24.8 ± 3.0*^,^^¶^	**<0**.**01**
Circumferential	−10.7 ± 1.6	−19.1 ± 2.2*	−19.7 ± 1.9*	−18.8 ± 2.1*	−18.5 ± 2.6*	**<0**.**01**
Longitudinal	−8.9 ± 1.2	−9.6 ± 1.4*	−11.4 ± 1.1*^,^^||^	−13.8 ± 1.2*^,^^||^^,^^¶^	−13.7 ± 0.9*^,^^||^^,^^¶^	**<0**.**01**
Time to peak (ms)						
Radial	357.6 ± 24.7	348.8 ± 22.4	345.1 ± 19.8	341.3 ± 22.4	338.2 ± 28.3	0.45
Circumferential	336.9 ± 25.1	323.1 ± 18.9	341.3 ± 20.0	335.5 ± 19.4	327.4 ± 29.5	0.34
Longitudinal	349.2 ± 18.6	345.5 ± 20.8	325.6 ± 21.1	344.8 ± 23.1	328.9 ± 20.7	0.37
Peak displacement (mm or °)						
Radial	2.6 ± 0.4	3.8 ± 1.1*	6.6 ± 0.9*^,^^||^	5.4 ± 0.6*^,^^||^^,^^¶^	5.9 ± 0.8*^,^^||^^,^^¶^	**<0**.**001**
Circumferential	3.5 ± 0.7	3.5 ± 0.9	−5.4 [−4.0,6.9]*	4.0 ± 1.1^¶^	−4.0 ± 1.3^¶^	**<0**.**01**
Longitudinal	4.8 ± 0.4	4.9 ± 0.9	8.2 ± 1.1*^,^^||^	5.9 ± 0.7*^,^^||^^,^^¶^	5.7 ± 0.9*^,^^||^^,^^¶^	**<0**.**001**

Bold numbers indicate a statistically significant difference.

Data are means ± standard deviations or median (interquartile range) as appropriate. RNM, remote non-infarcted myocardium.

* *P* < 0.01 for infarcted zone vs. other subgroups at peak radial, circumferential, and longitudinal strain.

^||^ *P* < 0.01 or *P* < 0.001 for peri-infarcted zone vs. other subgroups at peak strain and peak displacement.

^¶^ *P* < 0.01 or *P* < 0.001 for RNM in STEMI vs. other subgroups at peak strain and peak displacement.

Regarding synchronization, while the peak time of the infarcted zone, peri-infarct zone, and RNM varied slightly, there was no notable distinction between the STEMI group and the corresponding segments in the NC group (*P* = 0.45, 0.34, and 0.37, respectively).

The peak displacement variables also indicated that the radial displacement (2.6 mm ± 0.4 mm) (*P* < 0.001) and circumferential displacement (3.5° ± 0.7°) of the infarcted zone experienced significant reductions (*P* < 0.01) compared to RNM in the STEMI group. In the STEMI group, the longitudinal displacement of the infarcted area (4.8 mm ± 0.4 mm) and the peri-infarct zone (4.9 mm ± 0.9 mm) showed a significant decrease compared to the RNM (8.2 mm ± 1.1 mm) (*P* < 0.001) as indicated in Table 2.

The analysis at the segment level showed that out of 165 segments (46%), they were classified as both LGE^+^/MVO^+^, while 197 segments (54%) were classified as LGE^+^/MVO^−^. In comparison to LGE+/MVO-, the categories of RS, CS, and LS were all significantly impaired with percentages of 12.2% ± 2.1%, −9.6% ± 0.7%, and −6.8% ± 1.0%, respectively (*P* < 0.05). Refer to Table 3.

**Table 3:** Segmental strain parameters analysis between LGE^+^/MVO^+^ vs. LGE^+^/MVO^−^.

Variable	LGE^+^/MVO^+^	LGE^+^/MVO^−^	*P*-value
Segments *n* (%)	165 (46)	197 (54)	
Peak strain (%)			
Radial	12.2 ± 2.1	14.6 ± 3.2	**<0**.**05**
Circumferential	−9.6 ± 0.7	−10.8 ± 1.8	**<0**.**05**
Longitudinal	−6.8 ± 1.0	−9.2 ± 1.3	**<0**.**05**

Bold numbers indicate a statistically significant difference.

### 3.3. Dyssynchrony assessment

To further examine the correlation between LGE classification and strain, we categorized the infarcted segments into four groups based on the extent of transmural infarction. A total of 678 segments were measured with a degree ranging from 0% to 25%, while 65 segments had a degree between 26% and 50%. Additionally, there were 76 segments with a degree ranging from 51% to 75%, and 221 segments with a degree ranging from 76% to 100% (Table 4).

**Table 4:** Contraction reserve based on strain analysis following LGE classification.

LGE	Segments	Radial rebound stretch (δ1) (%)	Systolic septal delay (δ2) (ms)	Radial rebound displacement (δ3) (mm or °)	Circumferential strain stretch (δ4) (%)
0%–25%	678	5.9 (4.3, 8.7)	192 ± 16	2.6 (0.6, 3.8)	3.5 (1.4, 4.0)
26%–50%	65	5.9 ± 0.5	201 ± 27	1.8 ± 0.4*	3.5 ± 1.0
51%–75%	76	6.0 ± 0.4	188 ± 23	1.3 ± 0.2*^,^^¶^	3.4 ± 1.2
76%–100%	221	6.2 ± 0.4	148 ± 29*^,^^||^^,^^¶^	0.6 ± 0.2*^,^^||^^,^^¶^	−4.7 ± 1.4*^,^^||^^,^^¶^
*P*-value	–	0.67	**<0.01**	**<0.001**	**<0.01**

Bold numbers indicate a statistically significant difference.

Data are presented means ± standard deviations or median (interquartile range) as appropriate.

* *P* < 0.01 or *P* < 0.001 for infarct interval [76%–100%] vs. other subgroups at the variable of δ2, δ3, and δ4.

^||^ *P* < 0.01 or *P* < 0.001 for infarct interval [51%–75%] vs. other subgroups at the variable of δ2, δ3, and δ4.

^¶^ *P* < 0.01 or *P* < 0.001 for infarct interval [26%–50%] vs. other subgroups at the variable of δ2, δ3, and δ4.

No differences in radial rebound stretch were found between the four LGE^+^ groups (*P* = 0.67). The delay in systolic septum indicated that an infarction degree exceeding 75% was a significant threshold, and the rebound shrinkage of the septum (average, 148 ms) was considerably shorter in comparison to other infarction degrees (*P* < 0.01). The discoordination marker, measured from the radial rebound displacement, showed a significant decrease among the four grades (*P* < 0.001) with an increasingly severe transmural degree of infarction. Below 75%, there was no notable alteration in the stretch of circumferential strain; nevertheless, circumferential strain stretch reversed once it surpassed the boundary point (−4.7% ± 1.4%) (Table 4).

### 3.4. Reproducibility

Sixty-five patients were reanalyzed for the intra- and inter-observer feasibility and reproducibility. As shown in Supplementary Appendix Table S2, strain value had an excellent agreement between intra- and interobserver (ICC > 0.9).

## 4. Discussion

The study showed that strain, which is utilized to evaluate the LV systolic function, is a valuable tool for assessing dyssynchrony capacity in the ventricular septum after an infarction. The damaged septum oscillates in a two-peaked pattern, causing a decrease in the RS contractility of the affected heart muscle. Nevertheless, this modification of the strain becomes apparent when examining the analysis of the radial, circumferential, and longitudinal directions. A transmural degree of >75% represents the septal strain-contraction reserve cut-off point.

### 4.1. Morphology and function

During the acute phase, it was observed that individuals diagnosed with anterior STEMI exhibited reduced EF levels and elevated pulse pressure, indicating that myocardial damage impacts the contractility of the ventricles (22). Previous research has indicated that myocardial fibers play a crucial role in the contractile function of the myocardium. Cardiac output can be efficiently maintained by the distinctive twisting motion and arrangement of myocardial fibers (23, 24). The contractility of the left ventricle, which is mainly due to the myofibers, has a significant impact on the overall performance of the heart during the entire cardiac cycle (15). In our investigation, the longitudinal deformation was less than the recorded value mentioned in prior studies (25). Although there is a deviation in 2D strain on the long axis, it is still necessary to provide further evidence to demonstrate whether this represents an actual alteration in longitudinal function while preserving circumferential function. Nevertheless, there is no denying that this aspect of LV mechanics plays a crucial role in the ejection phase and significantly influences LV filling with its movement (26). In their study, Mangion et al. (27). demonstrated the significant impact of left ventricular blood flow on myocardial quality. The research involved a cohort of 55 patients, wherein individuals with a blood flow exceeding 600 ml/min exhibited a notable 10.2% augmentation in left ventricular mass when compared to those with lower blood flow levels. Buss et al. (28) performed a dobutamine stress analysis on a substantial sample of 2,585 CMR data, which revealed that inducible wall motion abnormalities were not indicative of the development of coronary artery disease. These studies highlight the numerous limitations associated with solely attributing impaired left ventricular contractility to global strain. The presence of edema and necrosis can adversely impact ventricular wall motion, underscoring the need for a more comprehensive analysis of strain vectors and identification of factors influencing contractile force.

### 4.2. Role of segmental strain alteration

In the STEMI group, there was a notable reduction in the radial, circumferential, and LS within the septal infarct region, indicating a decline in the movement of myocardial fibers in these three orientations as a result of myocardial necrosis. Simultaneously, both the peri-infarct zone and the RNM in remote areas were also individually impacted during the acute phase. According to a prior investigation, the RNM variant in individuals experiencing STEMI exhibited dynamic alterations over a period and displayed a strong association with the improvement of LVEF (29). As a result, CMR-FT has emerged as a superb method for measuring myocardial strain in patients with post-infarction. Follow-up examinations can thus also be performed directly with non-contrast cine sequences if, for example, certain patients are not allowed to receive contrast medium. Historically, there has been a dispute in the peri-infarct region and RNM concerning contractile function. Certain research has proposed the existence of compensatory enhanced cardiac performance (30), while others have indicated that this led to a decline in systolic function and structural irregularities (31). The structure of infarcted myocardium was pulled by the peri-infarct myocardium, which caused the changes in strain. These changes should be further investigated.

Considering the superposition of septal strain caused by the stretching from the peri-infarct myocardium, we considered the fact that the peak strain is influenced by both the myocardial contractile properties and the counteracting force exerted by afterload during systole (32). No significant variation in time to reach maximum value was noticed between the infarct septum, peri-infarct zone, and the RNM in the STEMI group when compared to the NC group (*P* = 0.45, 0.34, and 0.37, respectively). In the directionality of the strain vector, the interventricular septum exhibited a bimodal abnormal motion in both radial and circumferential directions. During the entire ventricular ejection process, the strain peak did not reflect myocardial performance at a fixed time. To overcome this limitation, it is necessary to conduct additional examination of the left ventricular systolic response to ventricular septal swing. The results of our study indicated that the interventricular septum exhibited varying degrees of rebound in radial and circumferential strains, leading to a reduction in the overall peak of strain (Figure 2).

### 4.3. Diagnostic value of strain dyssynchrony

Patients with anterior STEMI exhibited varying degrees of systolic elongation, which indicated ineffective contractions. The septum absorbed energy due to contractions in the LV free wall (32). According to previous research (33), the swinging motion of the septum results in unfavorable (inefficient) effort, which does not contribute positively to the functioning of the left ventricle. In our research, the LGE^+^ segments with myocardial infarction had varying degrees of septal swing. As described above, the ventricular septum and remote non-infarct myocardial contraction and stretching showed mechanical asynchrony characteristics. As per the Frank-Starling mechanism mentioned “an intrinsic adaptive response which serves to adjust each ventricular output to its inflow by increasing the force of contraction of the myocardium proportionally to any increase in the length of the muscle fibers” (34), when the infarcted myocardium surpasses the threshold of 75%, the mid-wall myocardium will no longer experience traction from the peri-infarct zone in this article. Instead, it will rotate the myocardium in the opposite direction to maintain a steady output of the left ventricle. Despite our and other observations (2, 33, 35, 36), all favoring that incorporating the effect of abnormal segmental conditions during dyssynchrony could enable quantification of peak strain, the physiological mechanism of ventricular septal swing needs to be further studied.

### 4.4. CMR feature tracking techniques

Similar to the previous studies (37) we have shown high diagnostic efficiency for MVO in STEMI. CMR-FT allows for multi-dimensional global and segmental myocardial strain assessment from standard cine images, without the need for specialized pulse sequences and additional scanning time (38). However, the reliability and accuracy of CMR-FT based strain analysis, more than strain-encoded MR (SENC), is dependent on reader experience. SENC provides a single heart-beat evaluation of myocardial strain with high reproducibility, especially in terms of regional analysis. SENC and feature tracking techniques are currently available for quantification analysis of myocardial deformation. Both techniques have advantages and drawbacks (39). Therefore, advances in cardiac imaging post-processing analysis are of great clinical importance in current practice.

### 4.5. Clinical implications

This research approach provides detailed data for understanding ventricular wall motion in patients with STEMI, which is complicated due to heart failure. Compared to EF, this is a more sensitive method to measure small changes in strain. The combination of CMR-LGE and strain puts forward an efficient risk stratification. This technique can help clinicians make accurate decisions, especially for patients with LBBB, avoiding the LV lead placed in the infarcted zone. The critical assessment of myocardial contractility obtained by CE-SSFP cine, is essential for patients with LBBB after ventricular septal infarction. Therefore, we suggest that patients with septal infarction should consider performing CMR scanning in the acute phase if the patient's condition permits.

### 4.6. Limitations

Several constraints of the study need to be addressed. Initially, in this research, the ventricular septal infarction occurred due to the blockage of the anterior descending artery. It could not be excluded that microvascular obstructions have an impact on the neighboring myocardium. Second, infarct size did not occupy all ventricular septum segments; hence, the strain results might be overestimated. Despite conducting a detailed examination of the composition and the extent of its spread, we would still require a more sophisticated algorithm to rectify the mistake automatically. The focus of this study was on the structural aspects of ventricular septal swing, and next we plan to conduct a more extensive examination of myocardial movement in forthcoming research. Additionally, the limited number of reperfused STEMI patients and the recruitment from a single center may have limited the applicability of our results. In addition, the foremost limitation of the current study is the lack of follow-up data, judging functional recovery and thus functional outcomes on a patient-by-patient basis.

## 5. Conclusions

CMR-FT strain analysis can entirely depict the asynchrony of remote non-infarcted myocardial motion caused by septal infarction. In anterior STEMI, the infarcted septum swings in a bimodal mode, and the injury myocardium reduces the RS contractility. A more than 75% transmural infarct degree would be the septal strain-contraction reserve cut-off point.

## Data Availability

The original contributions presented in the study are included in the article/**Supplementary Material**, further inquiries can be directed to the corresponding authors.
