# Supplementary material for: Left ventricular dyssynchrony measured by cardiovascular magnetic resonance-feature tracking in anterior ST-elevation myocardial infarction: relationship with microvascular occlusion myocardial damage
Source: Front Cardiovasc Med. 2023 Oct 10;10:1255063. doi: 10.3389/fcvm.2023.1255063 (PMC10602888; doi:10.3389/fcvm.2023.1255063)
Supplement: Supplementary file 1 [file Table1.docx]

Supplementary Material

Left Ventricular Dyssynchrony Measured by Cardiovascular Magnetic Resonance-Feature Tracking in Anterior ST-Elevation Myocardial Infarction: Relationship with Microvascular Occlusion Myocardial Damage

Zheng Sun^1,3†^, Yu Wang^2†^, Yingying Hu^4^, Fang Wu^1,3^, Nan Zhang^2*^, Zhi Liu^5^, Jie Lu^1,3^, Kuncheng Li^1,3*^

*** Correspondence:**

Nan Zhang, E-mail: zhangnan@ccmu.edu.cn; Kuncheng Li, E-mail: kunchengli55@gmail.com

**Online Appendix Table 1. Typical CMR parameters for T2w-STIR, CE-SSFP cine, and LGE acquisitions.**

| **Variable** | **T2w-STIR** | **CE-SSFP cine** | **LGE** |
| --- | --- | --- | --- |
| In-plane resolution | 1.3 × 1.3 | 1.4 × 1.8 | 1.4 × 1.9 |
| Slice thickness (mm) | 6 | 6 | 6 |
| TR (ms) | 2 RR interval | 2.84 | 904 |
| TE (ms) | 70 | 1.25 | 2 |
| ETL (n) | 17 | 1 | 1 |
| Bandwidth (KHz) | 235 | 1085 | 287 |
| Flip angle (°) | 180 | 50 | 20 |

**Online Appendix Table 2. The intra- and inter-observer variability of strain value.**

**R1-1**

| **Variable** |  | **ICC** | **95%CI** | | | ***P* value** | | |
| --- | --- | --- | --- | --- | --- | --- | --- | --- |
| **Intra-**  **Observer** | Peak strain | 0.96 | | 0.94 | 0.97 | | <0.05 |  |
|  | Time to peak | 0.92 | | 0.86 | 0.95 | | <0.05 |  |
|  | Peak displacement () | 0.93 | | 0.89 | 0.95 | | <0.05 |  |
| **Inter-**  **Observer** | Peak strain | 0.94 | | 0.91 | 0.96 | | <0.05 |  |
|  | Time to peak | 0.90 | | 0.85 | 0.92 | | <0.05 |  |
|  | Peak displacement | 0.91 | | 0.88 | 0.93 | | <0.05 |  |
